# Trackerless Augmented Reality Navigation for Robotic Partial Nephrectomy using Simultaneous Localization and Mapping

**DOI:** 10.21203/rs.3.rs-11271062/v1

**Published:** 2026-10-27

**Authors:** Jayender Jagadeesan, Emily Huang, Haoyin Zhou, Nathaniel Hansen, Nnamdi Ogugua Onochie, Timothy N. Clinton, Christopher P. Dall, Matthew Mossanen, Steven L. Chang

**Affiliations:** Brigham and Women’s Hospital; Brigham and Women’s Hospital; Brigham and Women’s Hospital; Brigham and Women’s Hospital; Brigham and Women’s Hospital; Brigham and Women’s Hospital; Brigham and Women’s Hospital; Brigham and Women’s Hospital; Brigham and Women’s Hospital

**Keywords:** nephrectomy, surgical navigation system, laparoscopy, augmented reality

## Abstract

**Purpose.:**

To evaluate the performance of a novel trackerless surgical navigation system for partial nephrectomy using 3D laparoscopy integrated with the simultaneous localization and mapping (SLAM) algorithm.

**Materials and Methods.:**

Patients undergoing robotic partial nephrectomy were retrospectively analyzed. From the preoperative CT/MR images, three-dimensional (3D) models of the kidney, tumor, and vasculature were generated using a deep learning-based approach. A calibrated stereo laparoscope acquired stereo video, which was processed using the SLAM algorithm to reconstruct high-fidelity 3D surface models of the kidney. This reconstruction was registered to the preoperative kidney model using anatomical landmarks and surface-based alignment. Augmented overlays of anatomy, including the tumor, were projected onto the surgical view. Accuracy was assessed using surface target registration error (STRE), landmark registration error (LRE), and localization error. A multi-institutional surgeon survey evaluated the trackerless navigation system using 10 representative cases.

**Results.:**

Twenty patients undergoing robotic partial nephrectomy were analyzed. Dense 3D surface reconstruction and laparoscope calibration were successful in all cases. Mean (SD; range) STRE, LRE, and localization errors were 1.47 (0.5; 0.6–2.5) mm, 4.3 (1.6; 1.1–6.5) mm, and 2.6 (1.2; 0.7–5.2) mm, respectively. Nine surgeons highly rated the 3D and 2D overlays (3.99 and 4.41/5). Reported cognitive load was moderate (2.38), with high scores for anatomical accuracy (4.44), trust in the overlay for intraoperative decision-making (3.89), and likelihood of adoption (4.56) on a 5-point scale.

**Conclusions.:**

A trackerless SLAM-based navigation system, evaluated retrospectively on recorded operative video, demonstrates the feasibility of accurate augmented visualization during partial nephrectomy.

## Introduction

Partial nephrectomy requires precise identification of critical anatomical landmarks and delineation of tumor margins, which can be challenging due to limited visualization, constrained access, and intraoperative deformation of key structures. The surgeon must rely on an understanding of anatomy, as well as the cognitive fusion of laparoscopic visualization with preoperative cross-sectional imaging, to target small renal masses during partial nephrectomy. Challenges with tumor identification, as is common with endophytic tumors, can lead to longer operative time and clamp time as well as increased risk of collecting system injury. Furthermore, inadequate targeting can increase the risk of positive margins and excess parenchymal loss^1,2^.

Numerous strategies have been employed to improve tumor localization during partial nephrectomy. Intraoperative intracorporeal ultrasound is widely employed because it can delineate tumor margins in real time and confirm the success of vascular clamping, while being easily integrated into the robotic platform^3^. Firefly vision with indocyanine green (ICG) uses nearinfrared fluorescence to enhance the contrast between renal parenchyma and tumor, though this approach is limited by poor ICG penetration and limited spatial resolution^4^. Some surgeons have also employed a preoperatively placed fiducial as a physical marker to guide tumor identification^5^.

Interest in augmented reality (AR) platforms with anatomic overlays has expanded significantly in recent years. Patient-specific navigation systems with AR displays for partial nephrectomy have demonstrated the feasibility and value of integrating preoperative CT-derived 3D anatomical models into the surgical workflow to improve intraoperative orientation. These platforms have enhanced understanding of tumor location, segmental arterial anatomy, and collecting system relationships, thereby supporting surgical planning and rehearsal, particularly for complex renal tumors. In some studies, such navigation approaches have also been associated with improved parenchymal preservation^6^. However, most existing systems rely on manual or semi-manual registration techniques^7–10^, external tracking hardware^11,12^, or fiducial markers^13^ to align preoperative models with intraoperative anatomy. Additionally, many systems depend on intraoperative markers or require extensive kidney exposure to facilitate registration^7–12,14–16^, assumptions that are not compatible with contemporary minimally invasive and regionalized surgical approaches that deliberately minimize organ manipulation. Together, these limitations highlight the need for fundamentally new, markerless and trackerless navigation paradigms capable of providing accurate, real-time guidance under realistic intraoperative conditions.

In this retrospective study, our objective was to evaluate the performance of a novel trackerless surgical navigation system for partial nephrectomy by applying it to recorded operative video from robotic partial nephrectomy procedures, leveraging 3D laparoscopy and simultaneous localization and mapping (SLAM) algorithms to provide accurate image overlays of anatomic landmarks that may guide the surgeon in tumor targeting and removal.

## Results

### Clinical Cohort and Patient Demographics

Data from twenty procedures in eligible patients were analyzed under an Institutional Review Board–approved protocol. The requirement for informed consent was waived given the retrospective nature of the study using recorded laparoscopic video data. Detailed patient tumor characteristics are summarized in Table 1.

Among the 20 patients who underwent surgery, 12 were male and 8 female, with a mean age of 57 years. The mean tumor size was 3.8 cm (range: 1.7–8.6 cm) based on preoperative imaging and 3.7 cm (range: 1.7–8.4 cm) based on final pathology. Eleven tumors were located in the right kidney. Of the twenty procedures performed, eight utilized a retroperitoneal approach while the others were resected using a transperitoneal approach. Notably, seven tumors were completely endophytic, and the majority of tumors were confirmed as clear cell renal cell carcinoma on pathological evaluation (see Table 1). Notably, one case resulted in a positive surgical margin. However, given the retrospective design of this study, the augmented navigation display was unavailable to the operating surgeon during resection. The procedure was performed using standard-of-care techniques without navigational assistance, underscoring the clinical gap this system aims to address and supporting the rationale for prospective deployment of the trackerless navigation system.

### Qualitative Assessment of Navigation Overlays

Figure 1 shows the results of the trackerless navigation system for 5 representative cases analyzed in this study. The trackerless navigation system successfully generated 2D augmented overlays of preoperative anatomical models onto intraoperative video across all analyzed cases, capable of running at 10–20 FPS. High-fidelity surface reconstructions of the exposed kidney surface were generated and co-registered to corresponding kidney surfaces generated from the preoperative CT/MR images (Fig. 1(c,f,i,k,m)). Furthermore, the registered anatomical models were overlaid on the laparoscopy videos to provide augmented visualization of the anatomy on the 2D live laparoscopy videos (Fig. 1(d)). Qualitatively, these overlays enabled clear visualization of tumor boundaries and surrounding anatomical landmarks, facilitating potential intraoperative orientation and target localization. The system demonstrated consistent performance across a range of tumor sizes, depths, and anatomical locations, including endophytic tumors, as shown in Fig. 1(g,i).

### Quantitative Performance Metrics

The system’s performance was evaluated using landmark registration error (LRE), surface target reconstruction error (STRE), and localization error metrics, as shown in Table 2. The mean LRE, STRE, and localization errors were 4.3 (1.6, 1.1–6.5) mm, 1.47 (0.5, 0.6–2.5) mm, and 2.6 (1.2, 0.7–5.2) mm, respectively. The average times for SLAM-based surface reconstruction, deep learning-based CT/MR segmentation, and landmark-based registration were less than 1 minute, 1 minute, and 5 minutes, respectively. The system maintained real-time performance for the 2D augmented visualization at approximately 10–20 frames per second, potentially enabling continuous intraoperative guidance without workflow disruption.

### Surgeon Survey Results

Nine surgeons from two teaching hospitals completed the survey, including seven attending surgeons (> 200 procedures each), one first-year fellow, and one fifth-year resident, both with experience as the primary operator in 10–50 cases. Overall, surgeon feedback indicated favorable perceptions of the augmented visualization system. On a 5-point Likert scale, both 3D augmented visualizations and 2D overlays received average ratings of 3.99 and 4.41, respectively. The mean cognitive load score across all respondents was 2.38 on a 5-point scale (1 = significant decrease in cognitive load; 5 = significant increase), suggesting that the navigation system could reduce the cognitive effort required for intraoperative decision-making. The mean ratings for overlay accuracy in identifying anatomical structures, trust in the overlay for intraoperative decision-making, and likelihood of adopting the technology in clinical practice were 4.44, 3.89, and 4.56 out of 5, respectively. Surgeons reported improved confidence in tumor localization and resection planning when viewing the videos. Most respondents expressed a high likelihood of adopting the system in clinical practice.

## Discussion

In this retrospective study, we evaluate a novel “trackerless” surgical navigation system that provides augmented visualization of the laparoscopic video integrated with 3D anatomical models derived from preoperative CT/MR imaging, with the potential to enable precise localization of renal tumors and delineation of their extent for complete resection. The system leverages standard-of-care stereo laparoscopic video to generate a high-fidelity dense 3D reconstruction of renal surface anatomy using novel SLAM algorithms. This reconstruction is then fused with preoperative imaging-derived models to produce an immersive, augmented visualization of the tumor and surrounding structures directly within the laparoscopic view.

A central advantage of this approach is that it does not require any external tracking hardware, such as optical or electromagnetic tracking systems. By relying exclusively on standard laparoscopic video data, the system avoids additional equipment costs, simplifies operating room setup, and minimizes disruption to established surgical workflows. To our knowledge, this represents the first trackerless navigation system to undergo initial human clinical evaluation for renal surgery, demonstrating the ability to support accurate tumor localization and margin delineation during partial nephrectomy.

Our results demonstrate accurate surface target registration between preoperative models and intraoperative 3D reconstructions, with a mean STRE of 1.47 mm. Furthermore, the system achieved a mean localization error of 2.6 mm when evaluating identifiable anatomical landmarks on 2D augmented laparoscopic views, indicating high fidelity alignment between the intraoperative scene and CT-derived models. Importantly, the total computational time required for surface reconstruction, model generation, and registration was under five minutes using an intuitive intraoperative software interface, supporting its feasibility for clinical deployment without prolonging operative workflow.

Notably, the system was successfully applied to seven completely endophytic tumors, where no visible surface landmarks were available to guide tumor localization. In these cases, the augmented visualization provided spatial context, enabling the user to infer tumor position and extent despite the absence of external anatomical cues. This highlights a key potential clinical advantage of the system, particularly for complex renal masses where traditional visual guidance is limited.

Beyond quantitative performance metrics, we conducted a structured surgeon survey to evaluate perceived accuracy, utility, and clinical applicability of the navigation system. Overall, surgeons reported a favorable assessment of the platform, rating both the 2D overlay and 3D augmented visualization highly on a 5-point Likert scale. Participants indicated that the system improved confidence in tumor localization and may reduce cognitive load by minimizing the need to mentally reconstruct 3D anatomy from 2D laparoscopic views. Importantly, all surveyed surgeons expressed a high likelihood of adopting the technology in future clinical practice, underscoring its perceived value and usability.

Despite these encouraging findings, several limitations should be acknowledged. First, although evaluated retrospectively using archived operative video data, the system has not yet been evaluated in a fully prospective, real-time interventional setting with outcome-based endpoints such as margin status, operative time, or renal functional preservation. Second, registration accuracy depends on the quality of both preoperative segmentation and intraoperative surface reconstruction, and errors in either component may affect final overlay precision. Additionally, tissue deformation during surgery remains a fundamental challenge in image-guided renal interventions, as changes in patient positioning, pneumoperitoneum, respiratory motion and surgical manipulation can introduce discrepancies between preoperative imaging and intraoperative anatomy.

Future work will focus on prospective clinical deployment, integration of deformable registration techniques, and incorporation of multimodal intraoperative imaging such as ultrasound to improve subsurface tumor localization. Expanding the system to account for dynamic organ deformation and validating its impact on oncologic and functional outcomes will be essential steps toward clinical translation.

## Methods

### Clinical Study Overview

With approval from the Institutional Review Board (Protocol number: 2024P000062), we conducted a retrospective analysis of laparoscopic video recordings and corresponding preoperative diagnostic CT and/or MR images from patients with renal masses suspicious for malignancy who underwent robotic partial nephrectomy at a single academic medical center. The requirement for written informed consent was waived because this study involved only secondary analysis of existing clinical data.

All patients underwent standard-of-care surgical procedures without any modification for the purposes of this study. Intraoperative laparoscopic video streams from both left and right stereo cameras were recorded directly from the laparoscopic tower. Cases performed using both transperitoneal and retroperitoneal approaches were included to ensure a representative sample of surgical techniques. No alterations to surgical workflow, instrumentation, or clinical decision-making were introduced as part of this analysis.

### CT/MR Imaging and Model Segmentation

Preoperative contrast-enhanced CT or MR images (Fig. 2 (A)) were imported into 3D Slicer, an open-source medical image processing software platform^17^. Imaging studies were selected within six months of surgery to ensure an accurate representation of intraoperative anatomy. Typical contrast-enhanced CT acquisition parameters included: 120 kVp, 150 mA tube current, 3 mm slice thickness, 0.9 x 0.9 mm in-plane resolution, and a three-phase contrast protocol. For contrast-enhanced MRI, the sequences used for segmentation were gradient echo images with TR = 4.02 ms, TE = 1.31 ms, 3 mm slice thickness, and a 320 × 182 acquisition matrix.

After resampling the images to an isotropic resolution of 1 mm, deep learning-based organ segmentation was performed using the nnU-Net-based *TotalSegmentator* module in 3D Slicer^18^ to automatically segment abdominal structures, including the kidneys, liver, spleen, aorta, and vena cava. Tumors were manually segmented using an intensity thresholding approach to ensure accurate delineation of the lesion voxels. All segmentation masks were subsequently converted into 3D surface models (Fig. 2 (B)) for integration and overlay onto the intraoperative video streams.

### SLAM-based 3D reconstruction of the Kidney

We implemented a customized *Simultaneous Localization and Mapping* (SLAM) framework to generate highly detailed 3D models of exposed kidney and tumor surfaces (Fig. 2 (D)) from stereo laparoscopy video streams (Fig. 2 (C))^19^. Our approach offers several key benefits designed for seamless integration into the surgical workflow:

- High-Quality Dense 3D Reconstruction: The algorithm uses an efficient stereo-matching algorithm to compute physical depth at every pixel. This results in a dense 3D model that accurately captures the morphology of the kidney and tumor, providing a superior visual reference compared to standard 2D laparoscopy.
- Large-Field Visualization: While laparoscopic views are often limited to small, localized regions, our SLAM system tracks camera motion to stitch together multiple views from video alone. This creates an extended, wide-area 3D map of the entire exposed organ, allowing the surgeon to maintain better spatial orientation during the procedure.
- Real-Time Intraoperative Performance: Optimized for fast processing, the SLAM method generates and updates 3D models and 2D augmented videos at 10–20 frames per second. This ensures that the augmented visualization remains synchronized with the live surgical maneuvers, a critical requirement for active navigation and decision-making. This capability is designed to ensure synchronization with live surgical maneuvers in prospective deployment. In the present study, however, all processing was applied to retrospectively captured video.
- Hardware-Free Integration: The system operates purely on the existing video data. By eliminating the need for external optical or electromagnetic trackers, the technology avoids adding complexity or bulk to the operating room, making it highly practical for routine clinical use.

In this study, the SLAM method was applied to retrospectively collected stereo videos from archived robotic partial nephrectomy cases. During the initial exposure phase of each recorded procedure, the surgeon performed a routine survey of the kidney surface as part of the standard operative technique. These archived video segments were subsequently provided to the SLAM algorithm, enabling it to incrementally fuse local scans into a cohesive, large-scale 3D model. This reconstructed surface serves as the critical bridge for registration, enabling preoperative CT data (including deep tumor margins and vascular anatomy) to be aligned with the operative anatomy. Once the CT-to-patient registration was established, the dense mapping module was deactivated, and the algorithm shifted focus to tracking the laparoscope position. In prospective deployment, this approach would support real-time delivery of augmented surgical guidance via 2D augmented video throughout the remainder of the procedure.

### Registration of Intraoperative Surface and Preoperative Models

To fuse or register the models, an expert surgeon selected four corresponding natural landmarks on both the reconstructed surface and the preoperative kidney models. These points enable an initial landmark-based registration^20^ that estimates the transformation between preoperative imaging and intraoperative surface coordinate spaces. The user then delineates a region of interest (ROI) around the exposed kidney on the reconstructed surface, which is used to crop both the reconstructed surface and the corresponding region in the CT-derived models. Surface-based registration is subsequently performed using iterative closest point (ICP)^21^ to refine the alignment between the cropped surfaces and determine the optimal transformation. This transformation is then applied to the preoperative 3D models to further align them with the intraoperative reconstructed surface, enabling accurate 3D augmented views of the kidney and tumor (Fig. 2 (E)), and 2D laparoscopy image overlaid with the models generated from the preoperative images (Fig. 2 (F)).

### Assessment metrics - reconstruction, registration and location errors

The accuracy of the trackerless navigation system was evaluated using three primary metrics: 1) Landmark Registration error (LRE) – the distance between corresponding landmarks selected on the preoperative imaging-derived model and the intraoperatively reconstructed surface after landmark-based registration; 2) Surface target registration error (STRE) – the point-to-point error between the cropped reconstructed surface and the cropped CT-derived surface model following ICP registration; 3) Localization error – the discrepancy between natural anatomical landmarks identified on the live 2D laparoscopic video and their corresponding locations on the augmented 3D model generated from the preoperative images. In addition, we recorded the computational performance of the system, including the time required for surface reconstruction, model generation from preoperative imaging, and both the initial landmark-based and the surface-based registration steps. All statistical analyses were exploratory and descriptive in nature. No formal hypothesis testing was performed. Quantitative metrics are reported as mean ± standard deviation (SD) and range.

### Surgeon surveys on realism and accuracy

We performed a survey of surgeons at two teaching hospitals to assess the perceived accuracy and clinical utility of augmented visualizations for guiding partial nephrectomy. The survey was administered via an online platform and included embedded videos from 10 randomly selected cases, each demonstrating both a 3D augmented visualization (Fig. 2E) and a corresponding 2D overlay (Fig. 2F). For each case, respondents rated the 3D augmented visualization and 2D overlay using a 5-point Likert scale (1 = very poor, 5 = excellent) and indicated their perceived utility for tumor resection (yes, no, or not sure). Participants also completed summary assessments evaluating: (1) the accuracy of the overlay in identifying anatomic structures, (2) the degree of trust in the overlay for intraoperative decision-making, (3) the impact on cognitive load, rated on a 5-point scale (1 = significantly decreases cognitive load, 5 = significantly increases cognitive load), and (4) the likelihood of adopting the technology in clinical practice, rated on a 5-point scale (1 = not likely at all, 5 = extremely likely).

## Supplementary Material

This is a list of supplementary files associated with this preprint. Click to download.

- TrackerlessNavigationPartialNephrectomy.mp4

## Figures and Tables

**Figure 1:**
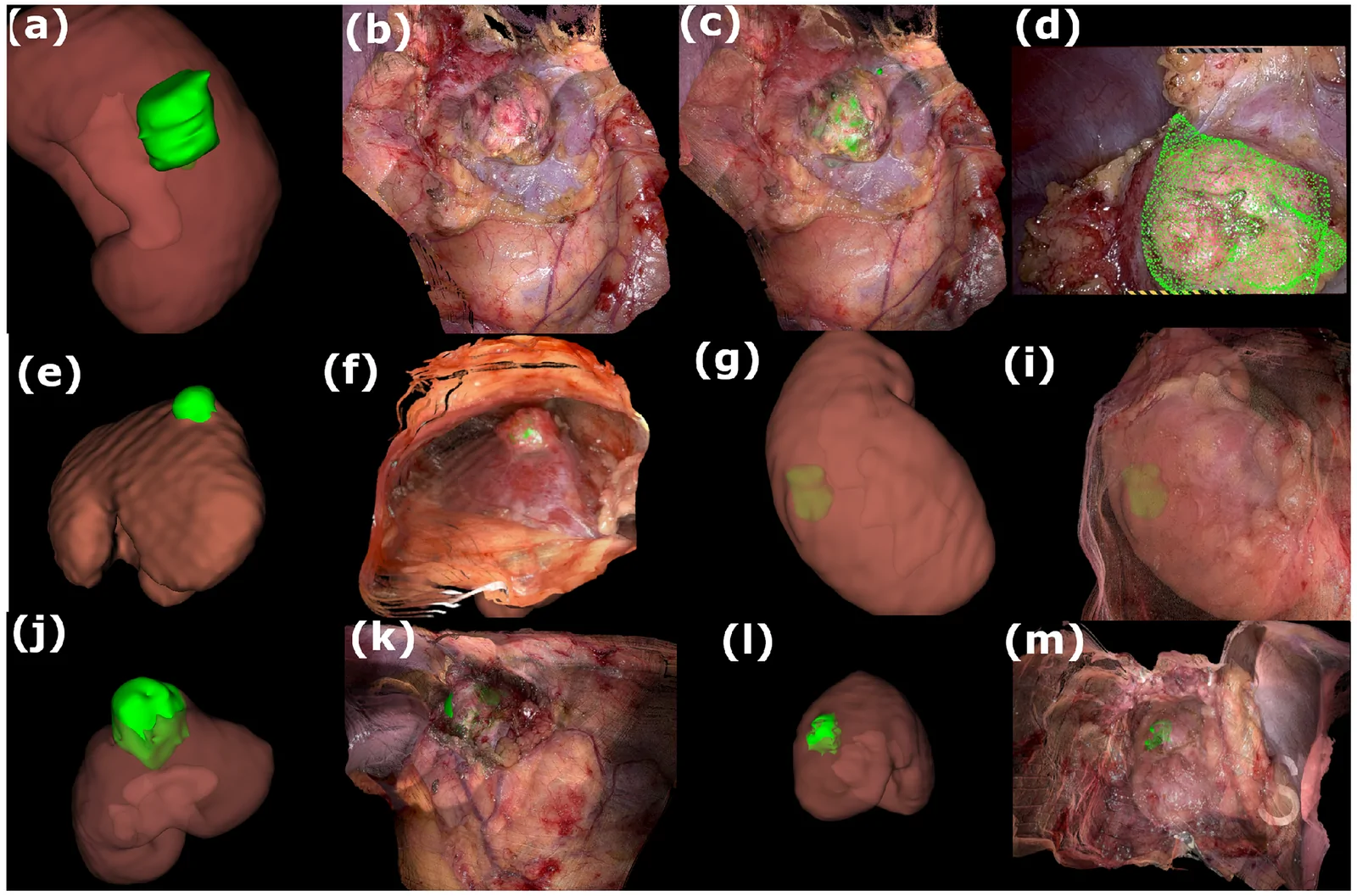
(a)-(d) Processing for a single case. (a) 3D model from preoperative images (b) Surface reconstruction from stereo laparoscopy image, (c) Overlay of surfaces from laparoscopy and CT models, (d) laparoscopy video with 3D model overlay. (e), (g), (j) and (l) 3D model segmented from preoperative CT/MRI, (f), (i), (k) and (m) Overlay of surfaces from laparoscopy and CT models.

**Figure 2:**
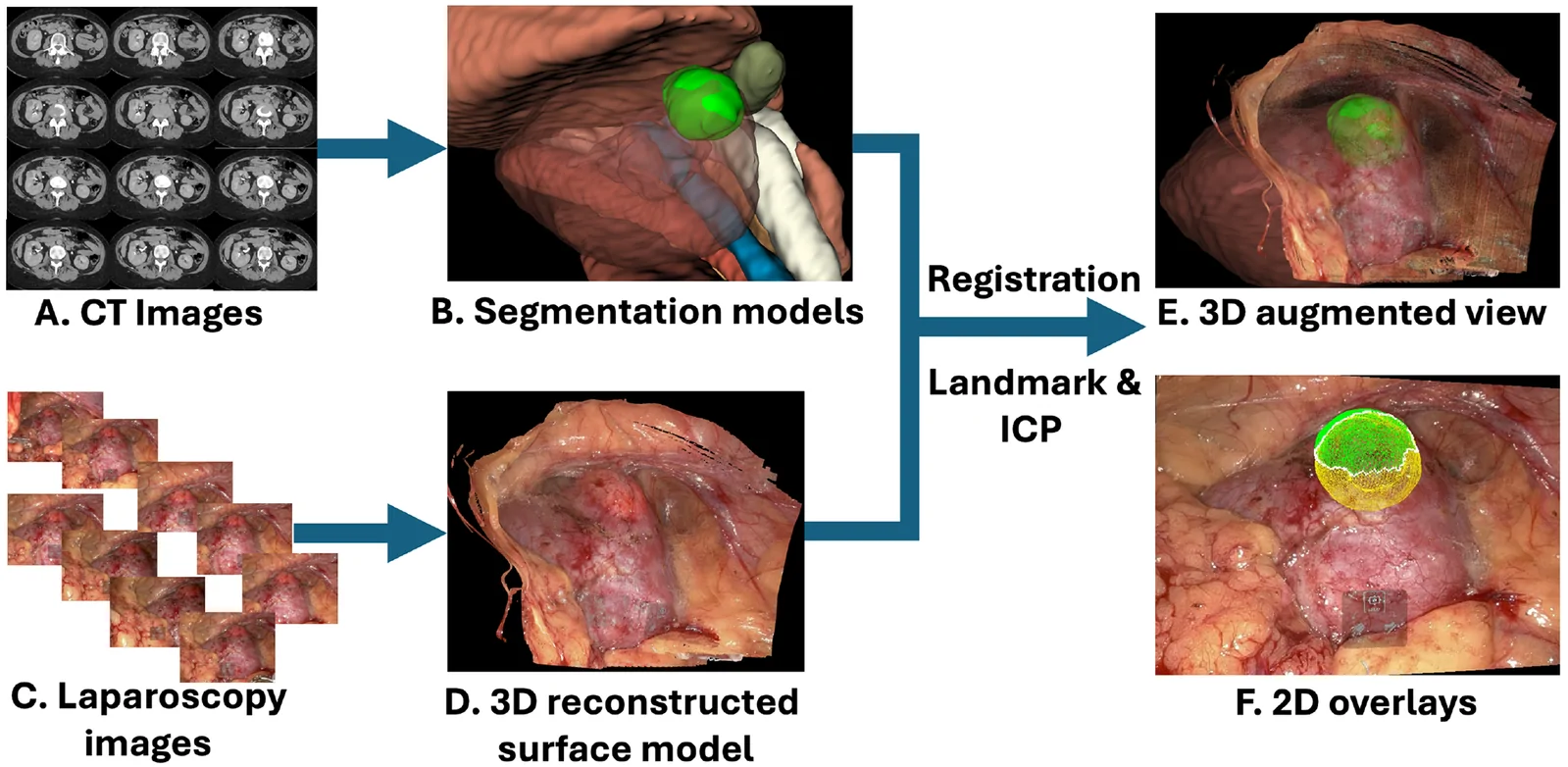
Diagnostic CT images of the patient (A) and patient-specific 3D anatomical models segmented from the diagnostic images (B). Following exposure of the kidney during robotic partial nephrectomy, a dense surface model (D) is reconstructed from intraoperative stereo laparoscopic video (C) using a SLAM-based approach. The preoperative and intraoperative models are co-registered to create the 3D augmented view (E) and 2D laparoscopy overlays (F). Semi-transparent rendering of the reconstructed surface enables visualization of subsurface tumor anatomy and surrounding structures overlaid onto the live laparoscopic view.

**Table 1:** Tumor characteristics.

Procedure	Left/Right	Tumor Size Based on Pre-Op Imaging (cm)	Tumor Size Based on Path Report (cm)	Endophytic (Y/N)	Transperitoneal (Y/N)	Pathology	Margin Status Positivity
1	R	2.9	3.2	N	Y	Clear cell (tubulopapillary) renal cell tumor	N
2	R	3.5	3.2	N	Y	Clear cell RCC	N
3	L	2.7	2.4	N	Y	Papillary RCC	N
4	L	3.1	4.3	Y	Y	Clear cell RCC	N
5	R	4	4.7	N	N	Clear cell RCC	N
6	R	4.8	3.5	Y	N	Clear cell RCC	N
7	L	5	4.3	N	Y	Angiomyolipoma	N
8	L	5.4	5.8	N	Y	Clear cell RCC	N
9	L	3.8, 2.7	4.5, 2.6, 0.8	N	Y	Papillary RCC, low-grade oncytic tumor, chromophobe RCC	Y
10	R	2.2	2.5	Y	Y	Clear cell RCC	N
11	L	5.4	5.3	Y	Y	Clear cell RCC	N
12	R	1.7	1.9	Y	Y	Clear cell RCC	N
13	L	6.8	N/A	N	N	Simple renal cortical cyst	N/A
14	R	1.8	1.8	N	N	Clear cell RCC	N
15	L	3.8, 3.9, 8.4	3.7, 4.0, 8.0	N	Y	Renal oncocytoma	N
16	R	4	3.8	Y	Y	Clear cell RCC	N/A
17	R	8.6	8.4	N	N	Hemorrhagic cyst	N/A
18	R	1.9	1.7	N	N	Papillary RCC	N
19	L	2	2.2, 2.0	N	N	Cystic clear cell RCC	N
20	R	2.2	2.2	Y	N	Clear cell RCC	N

**Table 2:** Quantitative errors for the cases - landmark registration, surface target registration and localization errors.

Procedure	Landmark Registration error (mm)	Surface target registration error (mm)	Localization Error (mm)
1	3.97	0.62	1.24
2	1.13	1.81	3.74
3	2.86	1.17	2.44
4	2.83	1.95	4.35
5	5.78	0.66	1.49
6	5.89	2.05	2.62
7	2.46	1.29	3.83
8	5.21	1.22	1.33
9	6.45	2.06	3.54
10	3.42	1.34	5.22
11	4.95	2.47	3.66
12	4.55	1.78	1.34
13	4.47	1.78	2.11
14	1.33	1.27	3.40
15	6.06	1.71	0.68
16	5.59	1.22	2.99
17	5.19	0.89	1.14
18	5.52	2.06	2.77
19	4.42	1.23	1.75
20	3.01	0.82	2.67

## Data Availability

None
